# Non-Functional Yet Terminal: A Case of Asymptomatic Retroperitoneal Paraganglioma With Distant Metastasis

**DOI:** 10.7759/cureus.43004

**Published:** 2023-08-05

**Authors:** Elvina C Lingas

**Affiliations:** 1 Hospital Medicine, Presbyterian Hospital, Albuquerque, USA

**Keywords:** neuroendocrine tumor, nonfunctional paraganglioma, rare cancer, adrenal pheochromocytoma, retroperitoneal paraganglioma, extra-adrenal paraganglioma

## Abstract

Retroperitoneal paraganglioma remains an extremely rare type of tumor that arises from either sympathetic or parasympathetic neural crest cells. It could be functional or non-functional. Non-functional paraganglioma may present as a diagnostic challenge since patients are usually asymptomatic and tend to present to the hospital with complications from the invasion of the tumor. Malignancy is usually determined by the degree of metastasis. The gold standard of diagnosis is biopsy and obtaining a sample for histological examination. This author presents a case of asymptomatic, non-functional retroperitoneal paraganglioma with distant metastasis.

## Introduction

Paraganglioma is a type of tumor that arises from neural crest cells in sympathetic or parasympathetic ganglion and can secrete catecholamines. Intra-adrenal paraganglioma is located in the adrenal and extra-adrenal paraganglioma and often arises from the head, neck, mediastinum, and retroperitoneum [1]. Although rare, it can also arise from other organs such as the pancreas [2], heart [3], and even lungs [4]. According to the updated 2022 WHO classification, pheochromocytoma is reserved for intra-adrenal paraganglioma and while paraganglioma may arise from both sympathetic and parasympathetic neurons, pheochromocytoma tends to represent a classical sympathetic form [5]. Paragangliomas are exceedingly rare [6]; however, they have been reported to have an increased incidence in the past two decades [7].

## Case presentation

A 74-year-old Navajo male with a past medical history including chronic atrial fibrillation with anticoagulation, type 2 diabetes mellitus, hypothyroidism, hypertension, and a history of CVA presented to the hospital with a one-week history of worsening flank pain. History was obtained via the patient and his caregiver. They stated that he had anorexia and weight loss although they were not sure for how long. He had been complaining of right flank pain for one week and had been taking over-the-counter (OTC) drugs with no relief. He stated that he had flank pain on and off, but it had never bothered him until now. They stated that they never sought care for this non-specific symptom since it usually went away after antacids and acetaminophen, leading them to think it was just acid reflux. He denied fever, chills, hematuria, diarrhea, nausea, or vomiting. He denied any history of palpitation, sweating, tremors, or frequent headaches. His social history was negative for smoking, alcohol, and illicit drugs.

The patient had normal vital signs. Initial laboratory markers showed a WBC of 9.9, hemoglobin (Hb) of 13.5, platelet of 323K, blood urea nitrogen (BUN) of 31, and creatinine of 2.05. His urinalysis showed 1 WBC and 1 RBC cell; no casts were present. His liver function tests were within normal limits. CT abdomen and pelvis without contrast demonstrated two large right retroperitoneal masses, multiple liver masses and bone lesions, atrophic left kidney with moderate hydronephrosis, and distal 13 mm x 9 mm ureter stone. CT with contrast was not performed due to his acute kidney injury upon presentation.

The patient was admitted and given IV fluid and his creatinine improved to 1.59. CT chest without contrast showed no pulmonary metastasis. Due to the high suspicion of cancer, interventional radiology (IR) was consulted to perform a biopsy. They requested an MRI abdomen with and without contrast which showed two previously seen right retroperitoneal masses inferior to the liver (sized >10 cm) and pressing on the inferior vena cava (IVC). Multiple necrotic hepatic masses were also imaged along with multiple retroperitoneal lymphadenopathies and multiple osseous metastases. IR biopsy of the right retroperitoneal mass was performed after his anticoagulation was held for 48 hours. Due to the atrophic left kidney and ureteral stone, urology was consulted; however, they felt that the patient’s presentation was more consistent with chronic obstruction, and no surgical intervention was recommended. His prostate-specific antigen (PSA) level and uric acid were within normal limits. His plasma metanephrine was elevated to 0.58. His serum chromogranin A was elevated to 69750 (normal range is <93 ng/mL). Fractionated metanephrine was not elevated.

Medical oncology was consulted. The pathology result was consistent with extra-adrenal paraganglioma. Due to the extent of the mass, consultation with the surgery team was recommended for debulking surgery; however, since the patient did not have symptoms from catecholamine excess, surgical intervention was not recommended. Palliative medicine also was consulted due to his advanced age and comorbidities. Since the patient had been stable throughout admission without significant pain and with stable laboratory markers, the oncology team recommended that he start palliative treatment as an outpatient which included lobenguane I-131 (Azedra), chemotherapy, and radiotherapy as well as consider locoregional therapies such as cryoablation and tumor embolization. However, due to the rarity of the disease, they recommended the patient be referred to an academic cancer center in a different state for further treatment. The patient was discharged at both follow-ups in stable condition. The patient was also referred for genetic testing. The post-discharge chart review documented that the patient was scheduled for radiation therapy. However, the patient passed away from some unknown cause and further chart review was not available since by that time, the patient had moved to a different state.

**Figure 1:**
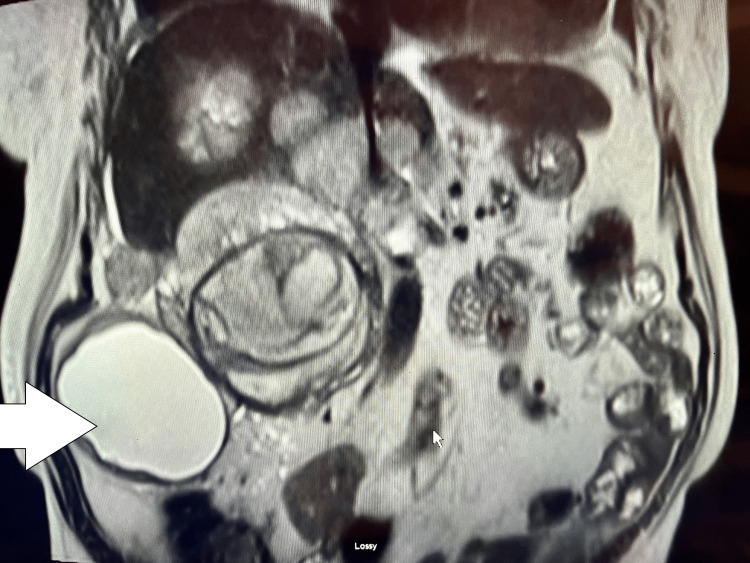
MRI image of the right retroperitoneal mass

**Figure 2:**
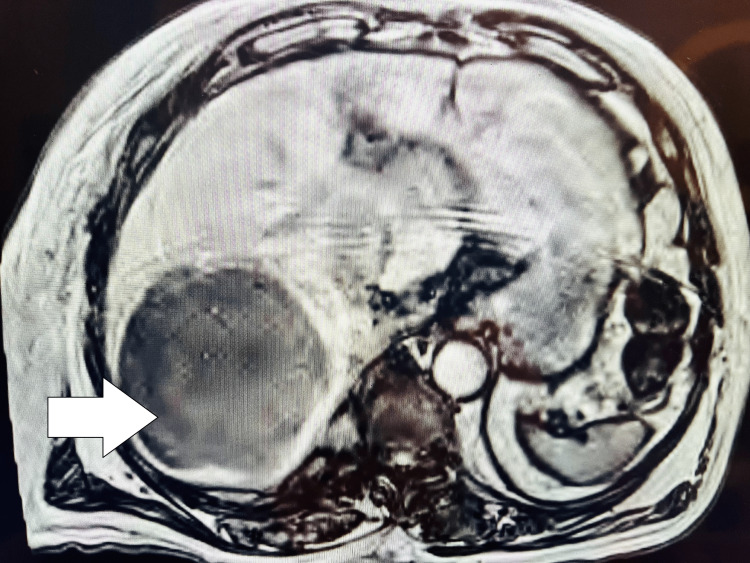
MRI image of necrotic liver metastasis

MRI images of retroperitoneal paraganglioma are shown in Figure 1 and liver metastasis in Figure 2.

## Discussion

Around 10%-15% of paraganglioma tumors are asymptomatic as it is with our case. These nonfunctional tumors are usually not diagnosed until they have metastasized or developed complications. This presents a diagnostic challenge since most patients with non-functional tumors only have nonspecific symptoms [8,9]. The complications can vary, but usually, it is related to local invasion, obstruction, and metastasis [6]. Our patient did have a history of atrial fibrillation and functional tumors can cause arrhythmia; however, this is unrelated to the tumor since his tumor was non-functional. Often the suspicion of malignancy is brought up by the sign of significant metastasis as it was with our patient. Metastasis sites are usually the lymph nodes, bone, and liver [10].

Our patient only had non-specific symptoms for an unclear duration; the recent change in his pain prompted him to visit the hospital. Significant metastasis was seen to the liver and bones, therefore, a chart review was performed to compare it with his previous imaging. However, he did not have any imaging of his abdomen area. His only CT was an angiogram of his head and neck upon his stroke diagnosis in 2019. The patient and his caregiver stated that his symptoms were minimal and usually went away with OTC treatment; therefore, they did not seek specialized care until this admission. There possibly is a component of racial disparity in seeking care as studies have documented racial disparity in cancer care and health literacy in the US [11].

The median year of diagnosis is usually age 40 which made our case atypical [9]. A large proportion of paraganglioma is hereditary due to Von Hippel-Lindau (VHL) gene mutations, multiple endocrine neoplasia type 2 (MEN2), neurofibromatosis type 1 (NF1), the Carney triad, and gene mutations of the subunits of succinate dehydrogenase (SDH) [12,13]. Despite being referred for genetic testing, we were unable to obtain the results.

Serum levels of chromogranin A are usually elevated with most neuroendocrine tumors which is consistent with our patient’s findings. CT or MRI is more sensitive than ultrasound; angiography can be used to detect small metastasis or to rule out vascular invasion [14]. Histology is the gold standard for diagnosis, and immunostaining is usually positive for neuroendocrine markers such as synaptophysin and chromogranin [15]. Surgical excision is the only curative treatment for localized disease. However, in the setting of metastasis, chemotherapy, radiation therapy, and/or locoregional treatment such as embolization may be recommended [16]. Surgery was consulted for our patient but he was not deemed a potential candidate. The prognosis of terminal-stage paraganglioma is very poor with less than a 40% five-year survival rate [17].

## Conclusions

Retroperitoneal paraganglioma tumors are exceedingly rare and have a poor prognosis. Non-functional tumors are often diagnosed late when it has metastasized. It is usually diagnosed by imaging, laboratory markers, and biopsy which is the gold standard. Treatment is usually surgical excision; however, in the event of non-excisable tumors with multiple metastasis, systemic chemotherapy, radiation therapy, and/or locoregional treatment aiming to reduce tumor burden may be recommended.
